# Dr. Maclean, on Tapping During Pregnancy

**Published:** 1802-03

**Authors:** James Moore

**Affiliations:** Grosvenor Street


### To Dr. BRADLEY.

Dear Sir,

I Received, a few days ago, from my brother, who is attached to the Embaſſy at Amiens, an Addreſs of a very intereſting nature, which was preſented to the Marquis Cornwallis. The original Inſtrument, and a Tranſlation which I have made, are encloſed, that you may publiſh either, or both, in your valuable Journal.

This Communication is not made on account of the additional proofs it contains in favour of the Inoculation of the Vaccine, all oppoſition to this great Diſcovery ſeems now to be ſilenced; like the doctrine of the Circulation of the Blood by the immortal Harvey, it is already eſtabliſhed: But it muſt give pleaſure to every humane mind, to be informed of the zeal with which this important improvement is received and propagated in France, where it is even made the ſubject of Congratulation to a Britiſh Embaſſador. It appears, that the name of Jenner is repeated there with the juſt eulogiums that are due to the man, who, practiſing medicine in a remote part of the country, had the ingenuity to raiſe out of an obſcure rumor, a moſt uſeful invention; who had the perſeverance to demonſtrate its innocence by convincing experiments; who, by ſuperior perior knowledge, refuted the objections of his enemies, and corrected the errors of his friends; and, laftly, who, fcorning to make a fecret of his difcovery for his private advantage, had the virtue to publifh it openly, for the Benefit of Mankind.

I am, &c.

*Grosvenor Street, Feb.* 5, 1802. JAMES MOORE.

---

LIBERTY. EQUALITY.

*Amiens, Frimaire 29th, 10th year of the Republic,* (16 *October*, 1801.)

From the MEMBERS of the JURY of HEALTH, and the MEDICAL COMMITTEE of the Department of the SOMME, to His EXCELLENCY the MINISTER PLENIPOTENTIARY of ENGLAND for the CONGRESS at AMIENS.

My Lord,

THE Jury are conftantly occupied with whatever relates to the prefervation of Man. Vaccination has juftly called forth their particular attention; and in the courfe of a year, a great variety of experiments have therefore been made here, upon more than fix hundred perfons. The firft Magiftrate of this Department has given every encouragement to our trials; and the Difcovery which has been made in your country, has been ftamped in our's with the feal of Infallibility. The Vaccine is now proved to be a Prefervative againft the Small-Pox. This can no longer be doubted. England, my Lord, has the honour of this Difcovery; we have received the Vaccine from your Phyficians. The Friends of Science never interrupt their fraternal intercourfe; and while their Governments wield the thunder of War, to decide their political contefts, men of Literature always remain in Peace. The Vaccine which has been fent here, has taken root; we have repeated the experiments of the immortal JENNER, and we have found them correct. Other experiments which we have inftituted, confirm the juftnefs of his conclufions, as well as what has been promulgated by Pearfon, Simmons, and Woodville; and the fortunate difcovery made in Gloucefterfhire fhall triumph for ever here. No improvement was ever affented to with fo much unanimity, in fpite of envy and avarice. But, my Lord, none of our experiments were more decifive than that which we fhall relate to you; not fo much for our gratification, as for the benefit of the art of Medicine and of Humanity; and in laying this before you, we wifh that the Glory which has been acquired may be tranſmitted to the DISCOVERER.

The 25th of laſt Germinal, (16 April) three infants at the Hoſpital St. Charles, named Duneuf Germain, Fracaſter, and Piſſon, who had before this been vaccinated with ſucceſs, were now inoculated with Small-pox matter: This produced no effect, and the triumph of the Vaccine was publiſhed. However, the incredulous and ill intentioned have always objections to raiſe, and calamities to prophecy; they aſſerted, that the preventive power was only temporary; that Jenner's proofs were not ſufficiently convincing; that that Phyſician lived far off; and, in ſhort, they denied every thing. But, upon the 25th Vendemiere laſt, (17th October) the Jury of Health at Amiens, inoculated theſe three children again. Every precaution was taken to prevent miſtakes, and they were in a manner deluged, rather than inoculated, with the moſt virulent Small-pox matter. Still, however, theſe children did not contract the Small-pox; they were not even diſordered; and now, at the expiration of ſixty days, they continue in perfect health. After this, who will dare to aſſert that the Vaccine is not a Preſervative againſt the Small-pox? There is no man, after ſuch authentic proofs, who would not bluſh to deny it. Will it yet be ſaid, that the preſervative power continues only for a time? Why then, after ſix complete months, ſhould the ſecond Inoculations with Small-pox fail? Why did they produce no more effect than the firſt? And, why is not the period pointed out, when the preſervative power ſhall ceaſe?

This is a difficult problem for the Detractors of Vaccination to ſolve. But they have changed their ground; they no longer deny the preſervative power which has been demonſtrated, they entrench themſelves behind certain accidents which they pretend have been occaſioned by the Vaccine; they have forged and publiſhed a hundred falſhoods, which have been quickly detected: The Jury of Health, my Lord, have the ſatisfaction of perceiving that they are too ſtrong for the Impoſtors: They are now of opinion that they ought no longer to reply to their intereſted opponents, who, although they are Phyſicians, tremble at every diſcovery favourable to humanity; they do not deſerve the honour of being confuted; they will never acknowledge their defeat, for they are neither influenced by truth nor the glory of the Art of Medicine, but are inſtigated ſolely by their deity, Avarice.

Accept, my Lord, our homage, and this account of the laſt experiments we have made, as an offering which we have the honour to preſent you with; they confirm, decifively, the admirable invention for which we are indebted to the Medical

Science

Science of England. We have already declared, that the French Phyficians have never ceafed to confider themfelves as brothers to your Phyficians; and, when you have finifhed your important labours at Amiens, the two nations will love each other reciprocally; and France and England, glorying in their valour, united by mutual efteem, fhall command Repofe to the reft of the World.

We have the honour to falute your Excellency with profound refpect.

SEVILLE.
CORNET.

